# Efficacy of walking exercise in promoting cognitive-psychosocial functions in men with prostate cancer receiving androgen deprivation therapy

**DOI:** 10.1186/1471-2407-12-324

**Published:** 2012-07-30

**Authors:** C Ellen Lee, Andrea Kilgour, YK James Lau

**Affiliations:** 1 Department of Physical Therapy, University of Manitoba, R106 – 771 McDermot Ave, Winnipeg, MB, R3E 0T6, Canada; 2 Department of Clinical Health Psychology, University of Manitoba, PZ350 - 771 Bannatynne Ave., Winnipeg, MB, R3E 3N4, Canada; 3 Eli Lilly Corporation, Lilly Corporate Center, Indianapolis, IN, 46285, USA

**Keywords:** Prostate cancer, Androgen deprivation therapy, Walking, Home-based exercise, Cognitive function, Psychosocial function

## Abstract

**Background:**

Prostate cancer is the most commonly diagnosed non-melanoma cancer among men. Androgen deprivation therapy (ADT) has been the core therapy for men with advanced prostate cancer. It is only in recent years that clinicians began to recognize the cognitive-psychosocial side effects from ADT, which significantly compromise the quality of life of prostate cancer survivors. The objectives of the study are to determine the efficacy of a simple and accessible home-based, walking exercise program in promoting cognitive and psychosocial functions of men with prostate cancer receiving ADT.

**Methods:**

A 6-month prospective, single-blinded, randomized controlled trial will be conducted to compare the Exercise Group with the Control Group. Twenty men with prostate cancer starting ADT will be recruited and randomly assigned to one of the two groups: the Exercise Group will receive instructions in setting up an individualized 6-month home-based, walking exercise program, while the Control Group will receive standard medical advice from the attending physician. The primary outcomes will be psychosocial and cognitive functions. Cognitive functions including memory, attention, working memory, and executive function will be assessed using a battery of neurocognitive tests at baseline and 6 months. Psychosocial functions including depression, anxiety and self-esteem will be assessed at baseline, 3 and 6 months using the Center for Epidemiological Studies Depression Scale, Spielberger State-Trait Anxiety Inventory, and Rosenberg Self-Esteem Scale*.*

**Discussion:**

The significance of the cognitive-psychosocial side effects of ADT in men with prostate cancer has only been recently recognized, and the management remains unclear. This study addresses this issue by designing a simple and accessible home-based, exercise program that may potentially have significant impact on reducing the cognitive and psychosocial side effects of ADT, and ultimately improving the health-related quality of life in men with prostate cancer receiving ADT.

**Trial registration:**

NCT00856102

## Background

In North America, prostate cancer is the most commonly diagnosed non-melanoma skin cancer. It is estimated that 25,500 men in Canada [1] and over 2.3 million men in the United States have prostate cancer [2]. More than one-third of men with prostate cancer will develop recurrent disease and receive androgen deprivation therapy (ADT) [3]. ADT is accomplished by bilateral orchiectomy or the use of a luteinizing hormone-releasing hormone agonist. The duration of response to ADT using hormone agonist ranges from 12 to 18 months, with 20% of patients having a complete prostate specific antigen response at 5 years [4]. However, there are a number of long-term health, cognitive, and psychosocial sequelae from ADT that significantly impact the health-related quality of life of men with prostate cancer.

The cognitive-psychosocial side effects of ADT are distinct health care needs of prostate cancer survivors that are not recognized until recent years. Studies have revealed that men receiving ADT have reduced cognitive function and self-esteem, and increased depression and anxiety secondary to the therapeutically-induced reduction of testosterone level [5-12]. Cognitive domains that are most significantly affected include verbal memory and learning [6,7], spatial memory and ability [5,8], visuomotor skills [10], executive function [6], and attention [7]. Men with prostate cancer receiving ADT are at a particularly higher risk of developing cognitive and depressive disorders than men without cancer [11]. These cognitive-psychosocial problems associated with ADT result in a significant reduction of health-related quality of life of prostate cancer survivors.

Physical activities through exercise is a lifestyle intervention that has protective effect against the risk of developing depression and anxiety disorders in the general population [13-15], and also improve cognitive function in older adults [16-18]. Regular exercise improves cardiovascular fitness, which in turn associates positively with cognitive performance in older adults and may have protective effect against cognitive decline later in life [19-23]. The underlying mechanism of how exercise improves cognitive and psychosocial functions are not clear. However, evidence suggests that increases in hippocampal volume [24,25] and brain monoaminergic systems [26] are possible mediating factors for improved memory function in individuals with higher cardiovascular fitness levels. Exercise, therefore, could have a potentially significant impact on improving the mental health and health-related quality of life of prostate cancer survivors.

Walking, in particular, is a simple and easily accessible form of exercise that can be readily incorporated into the long-term lifestyle of cancer survivors. Walking exercise has vast benefits on the quality of life and overall health including cognitive function in older adults [27,28]. In women with breast cancer, walking exercise has shown to significantly improve their health-related quality of life, fatigue, physical and mental health, [29-34] and even overall survival rate [35,36]. In men with prostate cancer on ADT, studies that investigated the role of exercise utilized mostly resistance exercise or a combination of resistance and aerobic trainings [37]. The results demonstrated that exercise could significantly reduce fatigue, improve muscular strength, physical function and overall health-related quality of life in men with prostate cancer. However, the effect of exercise, specifically walking, in the cognitive-psychosocial functions of men with prostate cancer on ADT is unknown. Therefore it is imperative to investigate if a simple walking exercise program will improve cognitive-psychosocial functions of men with prostate cancer on ADT.

The objective of the study is to determine the efficacy of a simple and accessible home-based walking exercise program in promoting cognitive-psychosocial functions of men with prostate cancer receiving ADT.

## Methods

A 6-month prospective, assessor-blinded, randomized controlled trial will be conducted to compare an Exercise Group to a Control Group. Patients will be recruited through CancerCare Manitoba. Baseline and follow-up visits will be conducted at the Department of Physical Therapy at the University of Manitoba. The University of Manitoba Research Ethics Board has approved the study protocol (Ethics Reference # H2008:318), which is also in compliance with the Helsinki Declaration.

### Participants

#### Inclusion criteria

Patients will be included in the study if they are men aged 50 years or older, diagnosed with adenocarcinoma prostate cancer, and will initiate and receive continuous ADT (luteinizing hormone releasing hormone agonist (LHRH) or combination of LHRH and anti-androgen) for at least 6 months after recruitment. Patients will be required to provide written informed consent to participate in the study.

#### Exclusion criteria

Patients will be excluded from the study if they have severe cardiac disease (New York Heart Association class III or greater), angina, pre-existing osteoporosis with T-score at or below −2.5, stable bone lesion, uncontrolled hypertension (blood pressure > 160/95 mm Hg), moderate to severe aortic stenosis, acute illness or fever, uncontrolled atrial or ventricular dysrhythmias, uncontrolled sinus tachycardia (> 120 beats per minute), third-degree atrio-ventricular heart block, active pericarditis or myocarditis, recent pulmonary embolism, deep vein thrombosis, uncontrolled diabetes, uncontrolled pain, cognitive impairment, history of falls due to balance impairment or lost of consciousness, or severe neuromusculoskeletal conditions that limit their ability to perform walking exercise (including ataxia, peripheral or sensory neuropathy, unstable bone lesion, severe arthritis, pathological lower limb fractures within 6 months, lower limb amputation).

#### Recruitment

A total of 20 patients will be recruited consecutively during office visits at the initial 3-month phase of the study at CancerCare Manitoba. The attending physician will screen the patients for inclusion criteria (men aged 50 or older, diagnosed with adenocarcinoma prostate cancer, will initiate and receive continuous ADT (LHRH or combination of LHRH and anti-androgen for at least 6 months). The physicians will be provided with an information sheet that provides brief information about study. If the patients meet the preliminary inclusion criteria, the physicians will provide a brief explanation about the study, and verbal consent to refer them to a clinical research nurse. The clinical research nurse will confirm that all inclusion and exclusion criteria are met, explain the study protocol, provide an information sheet for patients, and obtain written informed consent from the patients who would like to participate in the study. The clinical research nurse will provide the student research assistant (SRA) with the signed consent forms and contact information of the consented patients of the study.

### Assessment sessions

Each participant will attend a total of three assessment sessions: one baseline, and two follow-up sessions at 3 and 6 months after ADT initiation at the Department of Physical Therapy. The Exercise Group will complete telephone survey between follow-up sessions.

#### Baseline assessment session

The SRA, who is trained in health sciences field, will contact the participants to schedule a baseline assessment in coordination with a clinical psychology research assistant (CPRA), who is trained to administer neuropsychological tests.

The baseline assessment will be administered by the CPRA first, followed by the SRA. The CPRA will collect the sociodemographic characteristics, and administer the primary (cognitive-psychosocial functions) and secondary outcome measures (health related quality of life (HRQOL)). The SRA will collect data on the physical activity variables.

The CPRA will be blinded to the group assignments of the participants. After the baseline assessment, the SRA will assign the participant the first available number from a randomly generated list consisting of “1” and “2”, that are generated by mixed randomization, [38] using both simple randomization and permuted-block methods. Number “1” is designated as the Exercise Group that will receive instructions in setting up an individualized 6-month home-based walking exercise program in addition to their daily activities. Number “2” is designated as the Control Group that will be advised to perform daily activities as usual and follow the standard medical advice from their attending physician.

#### Follow-up sessions

The CPRA will schedule follow-up sessions for all participants at 3 and 6 months after ADT initiation. At 3 months, primary outcome (psychosocial functions) and secondary outcome variables (HRQOL) will be re-assessed. At 6 months, all primary (cognitive and psychosocial functions) and secondary (HRQOL) outcome measures will be re-assessed.

### Control group protocol

The SRA will provide each participant of the Control Group with a pedometer to monitor his ambulatory activities. The participants will also be provided with a take-home package that includes written instructions, pedometer daily logs, and self-addressed envelopes. The participants will be instructed to wear the pedometer during waking hours, except while bathing, swimming or any procedures that do not allow metal parts to be close to targeted treatment area. The pedometer will be worn on a waistband, at or slightly anterior to the mid-axillary placement. A 50-step preferred-pace trial walk will be used to determine the side of pedometer placement. The side that yields the least percentage error in step counts will be the side for the individual’s daily application [39]. At the end of each day, participants will record the date, total daily time wearing the pedometer, and the number of daily steps taken, and then reset the pedometer to zero. Every two weeks, the SRA will call the participants to check for concerns and remind them to return their pedometer daily logs using the self-addressed envelopes.

### Exercise group protocol

The SRA will assess the baseline health and fitness of the participants of the Exercise Group using an exercise screening form that includes the revised Physical Activity Readiness Questionnaire (PAR-Q)[40], and other screening questions for exercise contraindications and precautions. If any exercise contraindications (e.g. shortness of breath, severe headache, sudden onset of numbness or weakness) is present, the participant will be excluded from the study and referred to their family physician for investigation. If any exercise precautions (e.g. fever, severe cachexia, extreme fatigue, sickness or bone pain) is present, high-intensity or high-impact exercise will be avoided initially. If severe nausea or painful calf is present, the participants will be advised to consult their family physician for further investigation and clearance prior to beginning the exercise program.

The SRA will provide each participant of the Exercise Group with a pedometer and a take-home package that contains pedometer and exercise instructions and precautions, daily logs, general exercise tips, stretching instructions and self-addressed envelopes. The pedometer instructions and daily log instructions are same as per Control Group protocol.

The Exercise Group will follow a walking exercise program that comprises a progressive structured exercise protocol and progressive target daily step counts regimen. The structured exercise protocol will initially begin with a 10-minute walking session, 3 sessions per week. The Exercise Group participants will be instructed to perform a 5-minute warm-up period by walking slowly, followed by a 10-minute walking workout period, and then a 5-minute cool-down period by walking slowly and a stretching routine that is in the Exercise Group taken-home package. The SRA will also demonstrate to the participants how the stretches are to be done.

Participants will use the Borg Scale of Ratings of Perceived Exertion (RPE) [41] to estimate their exercise intensity level during the workout and warm-up/cool-down periods. RPE is a reliable method in monitoring and regulating exercise intensity, with high correlation to oxygen uptake (r ≥ 0.83) [42] and high internal consistency at different levels of velocities and heart rates (α > 0.90) [43]. During the warm-up/cool-down periods, the participants will maintain an exertion level between 7.5 and 8 on (i.e. extremely light level of exertion). During the 10-minute walking workout session, the participants will gradually increase their walking speeds until they attain and maintain an RPE level between 9 and 13 (i.e. very light to light exertion) according to their baseline physical activity level (sedentary, moderately active, or active) as reported on their self-reported physical activity questionnaire. Sedentary individuals will start at RPE level of 9 (very light exertion), moderately active individuals will start at RPE level of 11 (light exertion), and active individuals will start at RPE level of 13 (somewhat hard exertion).

Each participant’s exercise level will be progressed every two week, first in frequency (1-day increment), then walking session (5-minute increment, and lastly RPE level. Based on the set progression protocol, the Exercise Group participants will be performing five walking exercise sessions per week, 30 minutes walking workout period per session, and will have attained RPE level of 15 (heavy exertion) by around 18 to 20 weeks. This level of exercise protocol will be maintained for the rest of the study period. The participants are instructed to record the date, start time, exercise duration and maximum RPE attained during each session in an exercise daily log.

The participants will also be encouraged to walk at their chosen pace at times outside their structured exercise protocol so as to achieve their assigned target daily step counts. The initial target will be set at 5000 steps for sedentary individuals, 6000 steps for moderately active individuals, and 7000 steps for active individuals. The target daily step counts will be progressed every two weeks (1000-steps increment) up to a maximum daily target of 10,000 steps. The participants will be counseled on the warning symptoms of impending cardiovascular events including chest pain or discomfort, shortness of breath, dizziness, severe headache, sudden onset of numbness or weakness, or painful calf suggestive of deep vein thrombosis. They will be strongly advised to discontinue the exercise program and report these symptoms to their attending physician and SRA immediately.

The SRA will call the Exercise Group participants every two weeks to monitor their safety, adherence, and progress using a short, structured phone survey. The results of the phone survey will be used to guide the adjustments/progression of the structured exercise and step counts protocols. In addition, the participants will return their pedometer and exercise daily logs every four weeks.

### Data collection

At baseline session, the CRN will collect clinical data from the medical records of all consented patients that will include current cancer stage, time since diagnosis, treatment intent, testosterone level, prostate specific antigen level, Gleason score, comorbidity index. The SRA will collect sociodemographic data including age, marital status, ethnicity, living arrangement, education, work status, alcohol consumption and smoking history.

At baseline and 6-month follow-up visits, the primary outcome variable of *cognitive function* including memory, attention and working memory, and executive function will be assessed by the CPRA using well-established neurocognitive tests. Additionally, each participant will complete a standardized “effort test” to ensure validity of the test results. A minimum of 6 months between tests is required to prevent practice or carry-over effects. *Memory* will be assessed using: (1) the Brief Visuospatial Memory Test - Revised (BVMT-R) [44] for non-verbal memory, and (2) the California Verbal Learning Test, 2^nd^ Edition (CVLT-II) [45] for verbal memory. *Attention and working memory* will be assessed using: (3) the Digit Span and Letter-Number Sequencing tasks from the Wechsler Adult Intelligence Scale – 3^rd^ Edition (WAIS-III). [46]*Executive function* will be assessed using: (4) the Stroop Color and Word Test [47] for processing speed and inhibition, and (5) the Trail Making Test Parts A and B [48] for visuomotor tracking and mental flexibility. *Test-taking effort* of the neurocognitive tests will be confirmed using the (6) Word Memory Test (WMT) [49].

At baseline and all follow-up sessions (3 and 6 months), the primary (psychosocial functions) and secondary outcome variables (HRQOL) will be assessed. Cognitive function of *self-reported memory function* will be assessed using the Prospective Retrospective Memory Questionnaire (PRMQ) [50,51] for short and long-term memory. *Psychosocial function* will be assessed using: (8) the Center for Epidemiological Studies Depression (CES-D) for *depression, *[52] (9) the Spielberger State-Trait Anxiety Inventory (STAI) [53] for *anxiety*, (10) the Rosenberg Self-Esteem Scale (SES) for *self-esteem*. [54,55]*HRQOL* will be assessed using (12) the Functional Assessment of Cancer Therapy Prostate Module (FACT-P) [56] as a disease-specific global HRQOL instrument. Every four weeks, physical activity levels of both Control and Exercise groups will be monitored based on the completed pedometer daily logs returned by all participants.

#### Exercise group

*Exercise adherence* of the Exercise Group participants will be monitored using the exercise daily logs returned every four weeks, and phone surveys conducted by SRA every two weeks. The phone surveys will also be used to monitor *exercise safety and progress*.

## Statistical analysis

### Primary outcome variables

A mixed between-within-subjects multivariate analysis of variance (MANOVA) (Group X Time) will be conducted to determine if there is a significant interaction between the between-subjects (Group) and within-subjects (Time) comparisons for:

cognitive function based on the neurocognitive tests at baseline and 6-month follow-up session;

psychosocial function at baseline, 3- and 6-month follow-up sessions;

### Secondary outcome variables

Pearson product–moment correlation will be conducted to determine the association between HRQOL and primary outcome variables (cognitive and psychosocial functions).

For The Exercise Group, Spearman-Rank Or Pearson Product–Moment Correlations Will Be Performed To Determine If Baseline Sociodemographic, Clinical, Psychosocial, And Physical Activity Factors Are Significantly Associated With Exercise Adherence (Average Percentage Of Adherence To The Target Exercise Frequency, Duration And Intensity Based On The Exercise And Pedometer Daily Logs).

## Discussion

The significance of the cognitive-psychosocial side effects of ADT in men with prostate cancer has only been recently recognized, and the management remains unclear. Exercise through regular physical activity has proven to have tremendous health benefits in older adults and cancer survivors. However, the potential impacts of walking exercise on the cognitive and psychosocial functions of men with prostate cancer receiving ADT are unknown. The study addresses this issue by designing a simple and accessible home-based walking exercise program to determine its effect on promoting psychosocial and cognitive functions of men with prostate cancer receiving ADT. This study is significant because a successful home-based exercise program for men with prostate cancer would be cost-effective, and has a potentially significant impact on a number of ADT-related comorbidities, including reducing psychosocial and cognitive functional decline. This multidisciplinary study lays the foundation for the development of a prevention and wellness program within a multidisciplinary, comprehensive cancer care program for men with prostate cancer.

## Competing interests

None of the authors has any financial or non-financial competing interests to declare in relation to this manuscript. All authors were and are currently employed under organizations that do not, in any way, gain or lose financially from the publication of this manuscript. YKJL was involved in this study prior to employment at Eli Lilly Corporation.

## Authors’ contributions

CEL conceived the study, participated in the design of the study, coordinated acquisition of data, participated in data analysis and interpretation, drafted and revised critically the manuscript, and also provided final approval for the published version. ARK provided substantial support in the design of the study, acquisition of data, data analysis and interpretation of cognitive data, and also critically reviewed the manuscript and provided final approval for the published version. YKJL conceived the concept and design of the study, provided substantial support in the recruitment of patients, participated in data analysis and interpretation, drafted and revised critically the manuscript, and also provided final approval for the published version. All authors read and approved the final manuscript.

## Pre-publication history

The pre-publication history for this paper can be accessed here:

http://www.biomedcentral.com/1471-2407/12/324/prepub
